# Tissue Distribution Comparison of Six Active Ingredients from an Eucommiae Cortex Extract between Normal and Spontaneously Hypertensive Rats

**DOI:** 10.1155/2020/2049059

**Published:** 2020-06-07

**Authors:** Hejia Hu, Hongqin Xiao, Hongsong Bao, Mei Li, Cun Xue, YueTing Li, Guangcheng Wang, Siying Chen, Yong Huang, Lin Zheng, AiMin Wang, YongJun Li, ZiPeng Gong

**Affiliations:** ^1^State Key Laboratory of Functions and Applications of Medicinal Plants, Guizhou Provincial Key Laboratory of Pharmaceutics, Guizhou Medical University, 4 Beijing Road, Guiyang 550004, China; ^2^School of Pharmacy, Guizhou Medical University, 4 Beijing Road, Guiyang 550004, China; ^3^Guizhou Provincial Engineering Research Center for the Development and Application of Ethnic Medicine and TCM, Guizhou Medical University, 4 Beijing Road, Guiyang 550004, China

## Abstract

Eucommiae Cortex (EC), a rare, nourishing medicinal herb that is native in China, has good effect in the treatment of hypertension. In this study, we compared tissue distribution of six representative active components of EC extract—genipinic acid (GA), protocatechuic acid (PCA), neochlorogenic acid (NCA), chlorogenic acid (CA), (+)-pinoresinol di-O-*β*-D-glucopyranoside (PDG), and (+)-pinoresinol 4′-O-*β*-D-glucopyranoside (PG)—between normal rats and spontaneously hypertensive rats (SHRs). Each rat was intragastrically given EC extract at a dose of 5.4 g/kg. Rats were sacrificed at 10 min, 30 min, 2 h, and 8 h after administration; six rats were sacrificed at each time point. Then, we quickly harvested their major organs, including heart, liver, spleen, lungs, kidneys, and brain. Using ultraperformance liquid chromatography-tandem mass spectrometry (UPLC-MS/MS), we determined the levels of the above mentioned six components in the organs of both types of rats and then analyzed differences in the tissue distribution. The results showed that levels of each component differed between SHRs and the normal group at each time point. As time progressed, the number of organs in which GA distribution in each tissue of SHRs differed from that of the normal group gradually increased: SHRs showed lower GA levels than normal rats. Levels of PG and PDG in both groups at 10 and 30 min were similar. NCA and CA in the SHR group and the normal group at 10 min, 30 min, and 2 h were also similar to some extent. The results indicated that the pathological state of spontaneous hypertension could affect tissue distribution of EC active components in rats.

## 1. Introduction

Hypertension, known as the “silent killer” [1, 2], is a chronic systemic disease characterized by a continuous increase in systemic arterial blood pressure; it is one of the most common cardiovascular diseases in the world. Moreover, hypertension can cause complications such as encephalopathy and kidney disease. In recent years, with improvements in people's living standards and the faster pace of life, the number of hypertensive patients has been increasing [3]. Statistics show that there are more than 100 million hypertensive patients in China, and elderly patients account for 60%–70% of all patients [4]. Unfortunately, the blood pressure control rate of hypertensive patients is not satisfactory. At present, hypertension is mainly controlled and treated using Western medicine, such as calcium channel antagonists, *β*-blockers, and diuretics. Moreover, 90%–95% of hypertensive patients have spontaneous hypertension and must take medication for life. Therefore, side effects (such as liver function damage and gastrointestinal reactions) caused by long-term treatment with Western medicine alone should not be underestimated.

Traditional Chinese medicine has been found to have a unique advantage in the treatment of hypertension. In particular, some clinicians have found Eucommiae Cortex (EC) alone or in combination with Western medicine to have a significant effect on the disease [5]. EC is the dry bark of *Eucommia ulmoides* Oliver (also known as Sixian, Sizhong, or Kapok), which has a long history as medicine in China. EC has been shown to be efficacious in treating hypertension, tumors, and diabetes, among other diseases [6–8]. Researchers have discovered more than 100 chemical constituents of EC, including lignans, iridoids, phenolics, flavonoids, polysaccharides, and triterpenes. Lignans, iridoids, and phenolics are the important constituents contributing to the pharmacological efficacies of EC [9, 10]. (+)-pinoresinol di-O-*β*-D-glucopyranoside (PDG) and (+)-pinoresinol 4′-O -*β*-D-glucopyranoside (PG) are lignan compounds. Genipinic acid (GA) is an iridoid compound; protocatechuic acid (PCA), neochlorogenic acid (NCA), and chlorogenic acid (CA) are phenolic compounds. More importantly, PDG, GA, CA, and PCA have been shown to exhibit antihypertensive effects [11–13]. However, pharmacokinetic studies on the active ingredients of EC are still rare. In particular, the tissue distribution of the active antihypertensive components of EC in spontaneously hypertensive rats (SHRs) has not been reported, which to some extent precludes a deep understanding of this herb's therapeutic properties.

The tissue distribution of a drug is often used to evaluate its targets and determine whether the drug can accumulate in the body, as well as the extent of accumulation. It is only when the drug is distributed to target organs, target tissues, target cells, or other desired targets that it will demonstrate the best effect and the fewest toxic side effects. The main factors affecting drug distribution include the physicochemical properties of drugs and the physiological and pathological features of the various organs of the body. These factors lead drugs to be distributed differently between the different tissues of the body. Thus, research on drug distribution is crucial, especially in chronic diseases that require multiple long-term medications and typically raise safety issues (such as drug accumulation and toxic side effects).

In this study, we administered EC extract to both normal rats and SHRs to investigate the distribution of the herb's active components including GA, PCA, NCA, CA, PDG, and PG in important tissue organs of rats in the physiological state and in the pathological state of spontaneous hypertension that were administered with EC extract. We then analyzed the differences in distribution of these six constituents under different physiological and pathological conditions. Our data will provide a reference for the design of a long-term, rational, and safe drug dosing regimen for EC.

## 2. Materials and Methods

### 2.1. Instruments

We used an ACQUITY ultraperformance liquid chromatography (UPLC) I-Class Xevo TQ-S liquid triple-quadrupole mass spectrometry (MS) system with a MassLynx MS workstation (both from Waters, Milford, Massachusetts, US), a MRBP Noninvasive Blood Pressure Monitor (IITC, USA), an Allegra X-30R low-temperature, high-speed centrifuge (Beckman Coulter, Brea, California, US), a KQ-300DE ultrasonic cleaner (Kunshan Ultrasonic Instrument Co., Ltd., Kunshan, China), an MTN-2800D nitrogen drying device (Tianjin Aotesais Instrument Co., Ltd., Tianjin, China), a VX-III multitube vortex oscillator (Beijing Tajin Technology Co., Ltd., Beijing, China), an EL204 electronic balance (Shanghai Mettler Toledo Co., Shanghai, China), and a glass homogenizer (Shanghai Leigu Instrument Co., Ltd., Shanghai, China).

### 2.2. Materials

We obtained PDG, GA, CA, PCA, and puerarin (internal standard (IS)) (lot nos. 111537–201204, 111828–201403, 110753–201415, 110809–201205, and 110752–201514, respectively; purity: 90.9%, 94.6%, 96.2%, 99.9%, and 95.5%, respectively) from the National Institute for Food and Drug Control (Beijing, China). NCA (lot no: 160318; purity: 98%) was purchased from Sichuan Victory Biological Technology Co., Ltd. (Sichuan, China). PG (lot no: P09J7F8760; purity: 98%) was obtained from Shanghai Yuanye Bio-Technology Co., Ltd. (Shanghai, China). We purchased methanol (high-performance liquid chromatography (HPLC) grade) from Tianjin Kemiou Chemical Reagent Co., Ltd. (Tianjin, China). *E. ulmoides* cortex was purchased from the Guiyang Herbal Market (Guiyang, China). We have described the preparation of EC extract in our previous study [14]. The amounts of GA, PDG, PG, PCA, CA, and NCA in the EC extracts were 4.67, 10.39, 6.0, 0.78, 3.11, and 0.27 mg/g, respectively.

### 2.3. Animals

We purchased male, specific-pathogen-free Wistar rats and SHRs (220 ± 20 g) from Beijing Weitong Lihua Experimental Animal Technology Co., Ltd. (Beijing, China; animal license number: SCXK [Beijing] 2016–0006). All animal studies were approved by the Institutional Animal Care and Use Committee of Guizhou Medical University (no. 1702077), Guiyang, China.

### 2.4. Chromatographic and Mass Spectrometry Conditions

We used a Waters BEH C18 (2.1 × 50 mm, 1.7 *μ*m) column with a Waters Van Guard BEH C18 (2.1 × 5 mm, 1.7 *μ*m) guard column and an electrospray ionization (ESI) source. Scanning was performed in multireaction monitoring mode (MRM). Detailed parameters can be found in the literature [15].

### 2.5. Tissue Sample Processing Method

The tissue of each organ was accurately weighed and homogenized in a glass homogenizer with cool physiological saline based on weight (ratio of tissue to physiological saline, 1 g: 4 mL). We used the corresponding tissue homogenate as a blank homogenate, which we did not administer. The homogenate was sonicated for 5 min and centrifuged at 8000 revolutions per min (rpm) for 10 min. Then, we took 500 *μ*L of the upper homogenate, placed it into a 10 mL Eppendorf (EP) tube, and added 50 *μ*L of methanol to the tube. Next, we successively added 30 *μ*L of IS solution (100 ng/mL), 250 *μ*L of 1% formic acid, and 2 mL of methanol. After vortex mixing for 5 min, sonication for 10 min, and centrifugation (12,000 rpm for 10 min), we placed the supernatant into the EP tube and blew it dry under N_2_ (37°C) [16]. Next, we added 1 mL methanol into the tube for secondary precipitation. The tube was then vortex mixed for 5 min, sonicated for 10 min, and centrifuged at 12,000 rpm for 10 min. The supernatant was put into a new EP tube and blown dry under N_2_ (37°C). We then reconstituted the dried supernatant in 500 *μ*L of 50% methanol, vortex mixed it for 5 min, sonicated it for 10 min, and centrifuged it at 14,000 rpm for 10 min. Finally, we used the supernatant for injection analysis with UPLC–MS/MS.

## 3. Method Validation

### 3.1. Specificity

Blank tissue homogenate (500 *μ*L) of primary-organ tissues (heart, liver, spleen, lungs, kidneys, brain, stomach, intestines, muscles, and testes) from rats was processed according to the method described in “Tissue Sample Processing Method” above (without IS). We obtained a blank sample and used it for injection analysis of the A chromatogram. Certain concentrations of the reference solution and IS solution were added to the blank tissue homogenate and used for injection analysis. After administration, we took tissue homogenate of rats and used it for injection analysis.

### 3.2. Calibration Curves and Linearity

We began with a blank tissue homogenate (500 *μ*L) and sequentially added a series of mixed standard solutions containing the six components (50 *μ*L per solution) in order to prepare tissue homogenates with a series drug concentration according to “Tissue Sample Processing Method” above. The ratio of the peak area of the analyte to the IS (A/Ai) was taken as the ordinate, *y*. And linear regression was performed with each substance concentration (C) as the abscissa, *x*. The weighting coefficient was 1/*x*, and the linear equation obtained yielded calibration curves. The lower limit of quantification (LLOQ) for the six components was defined as the signal-to-noise ratio (S/N) ≥ 10.

### 3.3. Accuracy and Precision

In accordance with “Calibration Curves and Linearity” above, we prepared quality control (QC) samples of rat tissue homogenate at three concentrations: low, medium, and high. Each concentration was prepared in parallel as five replicates, and three samples were continuously injected over the course of the day. We continuously measured different concentrations for 3 days and separately calculated intraday precision and interday precision for the method.

### 3.4. Extraction Recovery and Matrix Effects

We began with a blank tissue homogenate (500 *μ*L) and prepared QC samples of the six-component tissue homogenate according to “Calibration Curves and Linearity” above, with each concentration prepared in five parallel replicates. Sample A was prepared according to “Tissue Sample Processing Method” above. We then took another 500 *μ*L blank tissue homogenate but did not add the mixed standard solution; otherwise, we followed “Tissue Sample Processing Method” above. Mixed standard solutions of the above concentrations (each concentration was prepared in five parallel replicates) were added to the supernatant obtained after centrifugation, the supernatant was blown dry under N_2_ (37°C), and then the dried supernatant was reconstituted in 500 *μ*L of 50% methanol to obtain sample B. We then took a mixed standard solution (low, medium, and high concentrations) with IS and blew it dry. The residue was reconstituted in 500 *μ*L of 50% methanol to obtain sample C. The extraction recovery was the ratio of the peak area of sample A to that of sample B. The matrix effect was the ratio of the peak area of sample B to that of sample C.

### 3.5. Stability

We prepared blank tissue homogenate as high-concentration QC samples according to “Calibration Curves and Linearity” above. Then, we left the samples at room temperature for 24 h, and then we froze them (−80°C) for 24 h. We repeated this freeze/thaw cycle three times. Concentrations of the processed samples were measured to investigate the stability of the six components in the tissue homogenate at room temperature, when frozen, and after repeated freeze and thaw. We prepared each concentration in parallel to obtain five replicates.

### 3.6. Tissue Distribution Study

The blood pressure of Wistar rats and SHRs was measured before the tissue distribution experiment. Qualified SHRs were selected for the tissue distribution experiment if the average value of the 3 systolic blood pressure (SBP) values was ≥150 mmHg. The pressure measurement method was as follows: the rats were placed in a 37°C preheated chamber for 10 min before the pressure measurement; then the rat tail artery SBP was measured with an MRBP noninvasive sphygmomanometer. The three stable SBP values of a rat in the resting state were recorded, and the average value of SBP was obtained as shown in Table 1.

After preparing them for the experiment, we divided our 24 male Wistar rats and 24 qualified SHRs (220 ± 20 g) into four groups, for the four time points of 10 min, 30 min, 2 h, and 8 h. Rats fasted for 24 h before administration of the extract, drinking water freely. We orally administered an EC extract to rats at a dose of 5.4 g/kg. At 10 min, 30 min, 2 h, and 8 h after administration, rats were sacrificed, and their hearts, livers, spleens, lungs, kidneys, brains, stomachs, small intestines, muscles, and testes were quickly excised. We washed away blood and contents of the tissue surface using ice physiological saline and dried the organs with a filter paper. The tissue was then weighed, placed in a valve bag, and stored at −80°C until analysis.

## 4. Results

### 4.1. Method Validation

#### 4.1.1. Specificity

**Figure 1:**
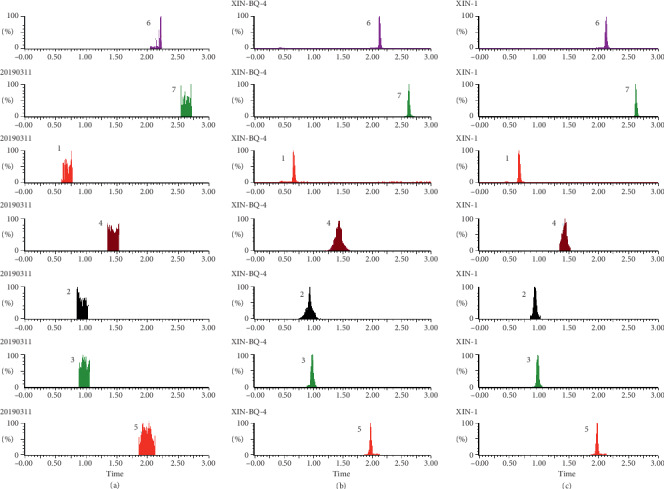
Typical UPLC-MS chromatograms of rat heart. (a) Blank heart sample, (b) a heart sample spiked with standard compounds, and (c) a heart sample obtained from a rat after oral administration of EC extract. (1) GA, (2) NCA, (3) PCA, (4) CA, (5) IS, (6) PDG, and (7) PG.

**Figure 2:**
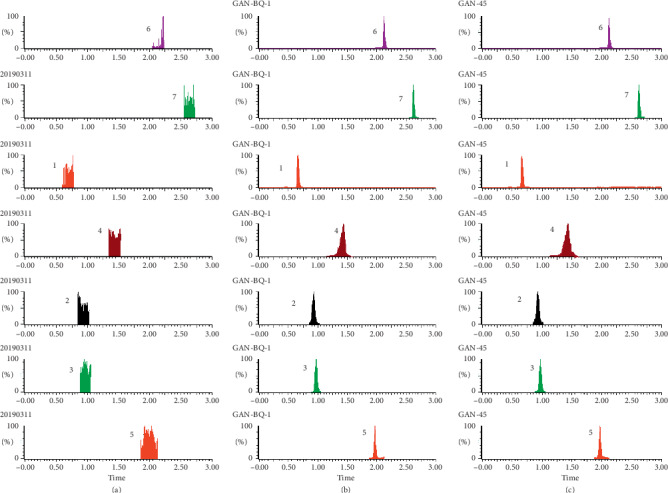
Typical UPLC-MS chromatograms of rat liver. (a) Blank liver sample, (b) a liver sample spiked with standard compounds, and (c) a liver sample obtained from a rat after oral administration of EC extract. (1) GA, (2) NCA, (3) PCA, (4) CA, (5) IS, (6) PDG, and (7) PG.

**Figure 3:**
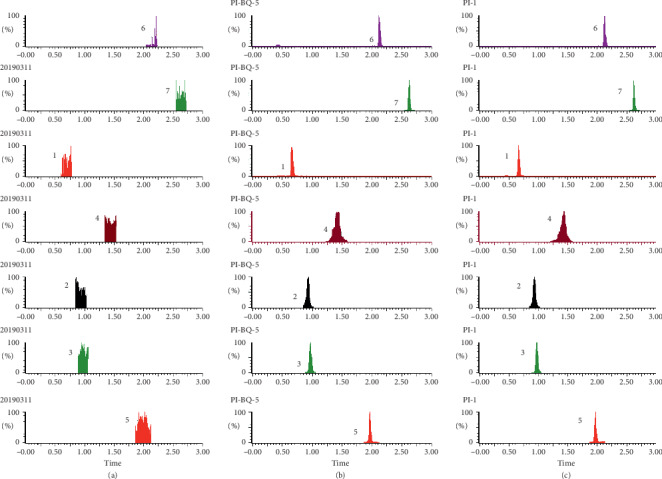
Typical UPLC-MS chromatograms of rat spleen. (a) Blank spleen sample, (b) a spleen sample spiked with standard compounds, and (c) a spleen sample obtained from a rat after oral administration of EC extract. (1) GA, (2) NCA, (3) PCA, (4) CA, (5) IS, (6) PDG, and (7) PG.

**Figure 4:**
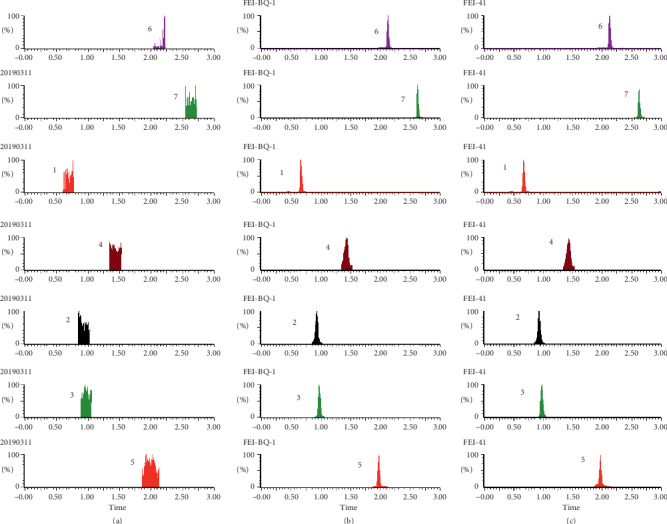
Typical UPLC-MS chromatograms of rat lung. (a) Blank lung sample, (b) a lung sample spiked with standard compounds, and (c) a lung sample obtained from a rat after oral administration of EC extract. (1) GA, (2) NCA, (3) PCA, (4) CA, (5) IS, (6) PDG, and (7) PG.

**Figure 5:**
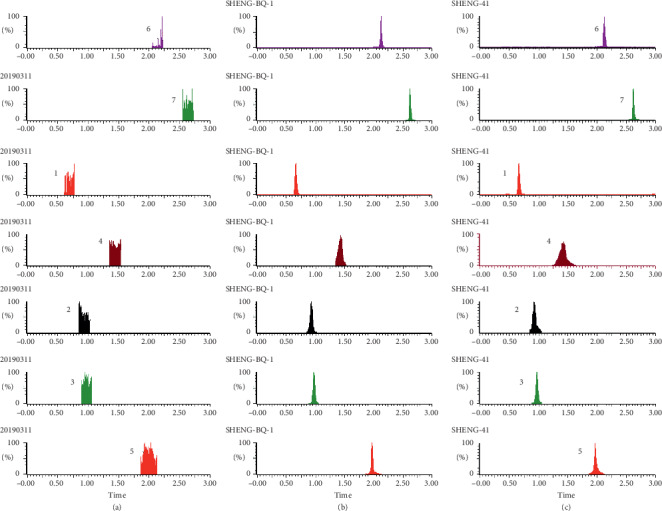
Typical UPLC-MS chromatograms of rat kidney. (a) Blank kidney sample, (b) a kidney sample spiked with standard compounds, and (c) a kidney sample obtained from a rat after oral administration of EC extract. (1) GA, (2) NCA, (3) PCA, (4) CA, (5) IS, (6) PDG, and (7) PG.

**Figure 6:**
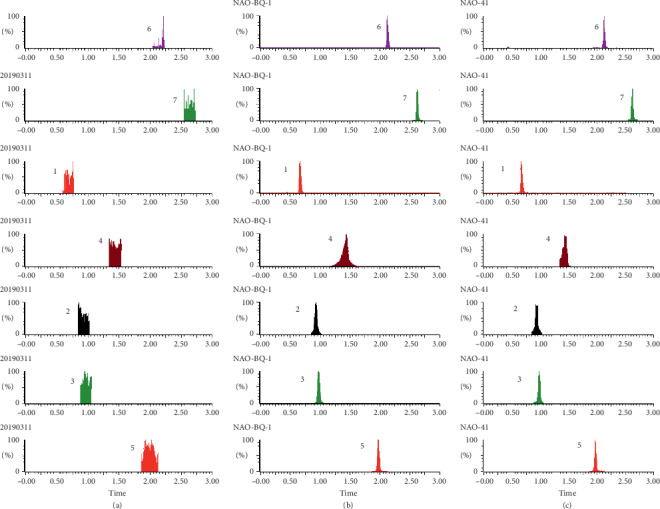
Typical UPLC-MS chromatograms of rat brain. (a) Blank brain sample, (b) a brain sample spiked with standard compounds, and (c) a brain sample obtained from a rat after oral administration of EC extract. (1) GA, (2) NCA, (3) PCA, (4) CA, (5) IS, (6) PDG, and (7) PG.

**Figure 7:**
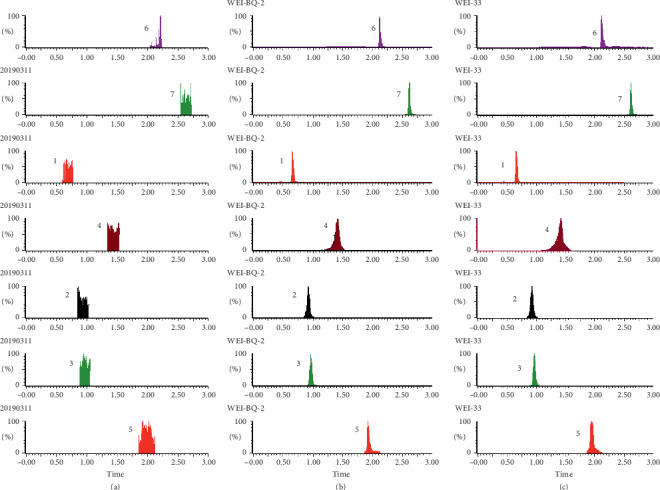
Typical UPLC-MS chromatograms of rat stomach. (a) Blank stomach sample, (b) a stomach sample spiked with standard compounds, and (c) a stomach sample obtained from a rat after oral administration of EC extract. (1) GA, (2) NCA, (3) PCA, (4) CA, (5) IS, (6) PDG, and (7) PG.

**Figure 8:**
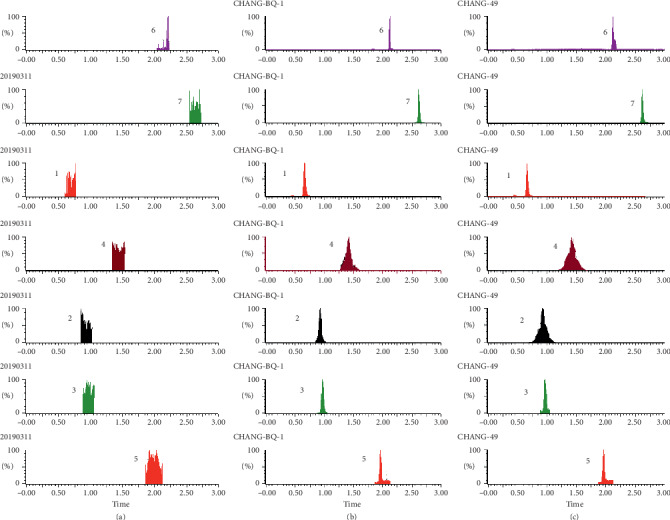
Typical UPLC-MS chromatograms of rat intestine. (a) Blank intestine sample, (b) an intestine sample spiked with standard compounds, and (c) an intestine sample obtained from a rat after oral administration of EC extract. (1) GA, (2) NCA, (3) PCA, (4) CA, (5) IS, (6) PDG, and (7) PG.

As shown in Figures 1–8, under the established conditions of UPLC-MS/MS, the blank rat tissue homogenate sample, rat tissue homogenate sample spiked with standard compounds and IS, and tissue homogenate sample obtained from a rat after oral administration of EC extract, the six components of EC extract separated well. There were no impurity interference in the blank tissue homogenate, the blank tissue homogenate that was added to the mixed standard solution and IS, or the rat tissue homogenate sample.

#### 4.1.2. Calibration Curves and Linearity

As shown in Tables 2 and 3, the six components in rat tissue homogenate had good linear relationships in the linear range, and the correlation curve (*R*^2^) of each component was >0.99.

#### 4.1.3. Precision and Accuracy

We assessed intraday and interday precision of the six components in the rat tissue homogenate at low, medium, and high concentrations. The results showed that the relative standard deviation (RSD; %) of both intraday and interday component precision ranged from 1.05% to 14.52%. Furthermore, the accuracy range was 85.57%–113.94% for the medium and high concentrations and 82.65%–117.35% for the low concentration. These data suggested that the method was accurate, reliable, and reproducible.

#### 4.1.4. Extraction Recovery and Matrix Effect

We investigated the extraction recovery rate and matrix effect of the high concentration of tissue homogenate samples in the linear ranges of the six components. The rate of extraction recovery ranged from 82.35% to 106.54%, while the matrix effect ranged from 84.12% to 106.54%.

#### 4.1.5. Stability

We investigated the stability of the six components in rat tissue homogenate left at room temperature (about 20°C) for 24 h, frozen (−80°C) for 1 month, and frozen and thawed for three cycles. The results showed that the homogenate samples were stable at room temperature for 24 h, −80°C for 1 month, and after three repetitions of the freeze-thaw cycle. Accuracy ranged from 82.16% to 114.63%, and the RSD was between 1.69% and 13.88%.

#### 4.1.6. Tissue Distribution of Six Active Components of EC Extract in Normal Rats and SHRs after Administration

**Figure 9:**
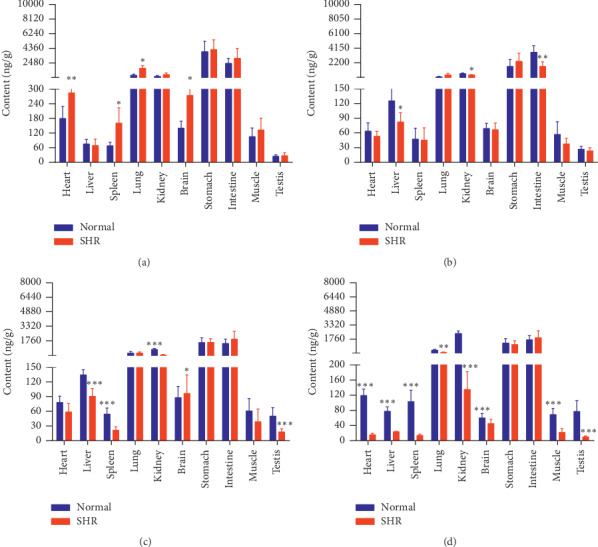
Content of GA in different rat tissue homogenates of the normal and SHR models at four different points in time after intragastric administration of EC extract (mean ± SD, *n* = 5). Relative to the normal group, ^*∗*^*P* < 0.05, ^*∗∗*^*P* < 0.01, and ^*∗∗∗*^*P* < 0.001 (the same applies to the following tables). (a) 10 min, (b) 30 min, (c) 2 h, and (d) 8 h.

**Figure 10:**
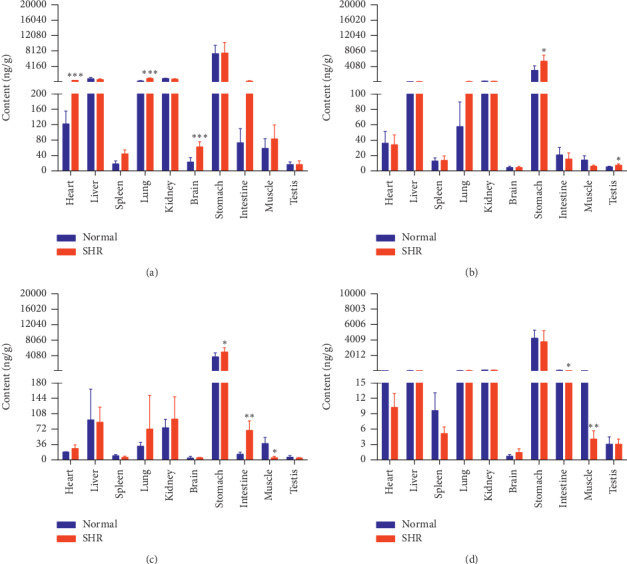
Content of PCA in different rat tissue homogenates of the normal and SHR models at four different time points after intragastric administration of EC extract (mean ± SD, *n* = 5). (a) 10 min, (b) 30 min, (c) 2 h, and (d) 8 h.

**Figure 11:**
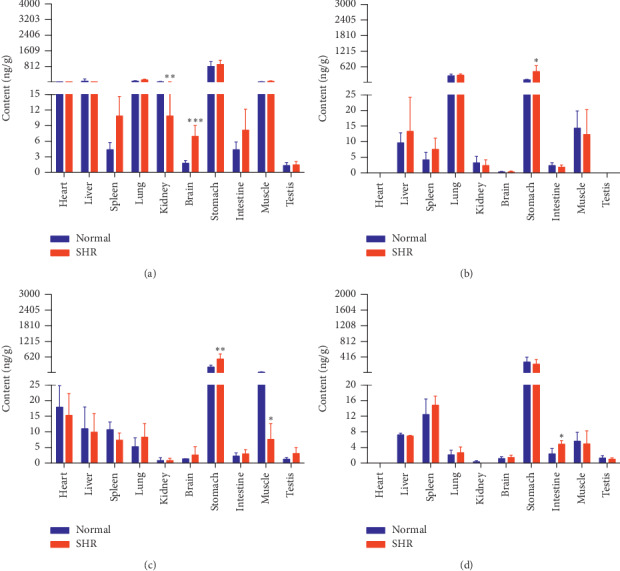
Content of NCA in different rat tissue homogenates of the normal and SHR models at four different points in time after intragastric administration of EC extract (mean ± SD, *n* = 5). (a) 10 min, (b) 30 min, (c) 2 h, and (d) 8 h.

**Figure 12:**
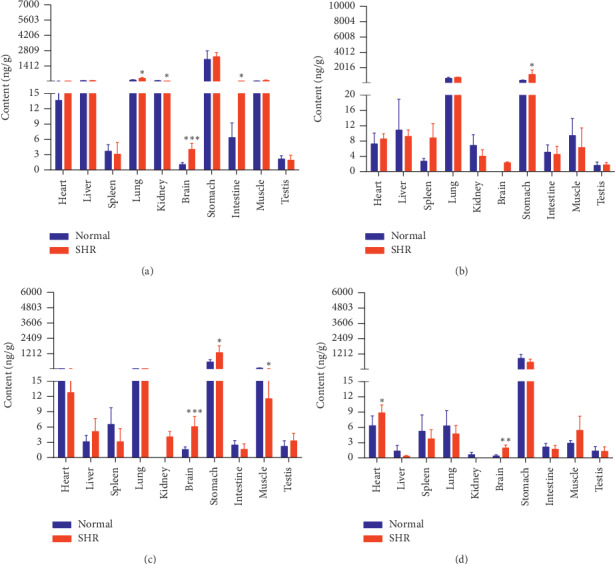
Content of CA in different rat tissue homogenates of the normal and SHR models at four different points in time after intragastric administration of EC extract (mean ± SD, *n* = 5). (a) 10 min, (b) 30 min, (c) 2 h, and (d) 8 h.

**Figure 13:**
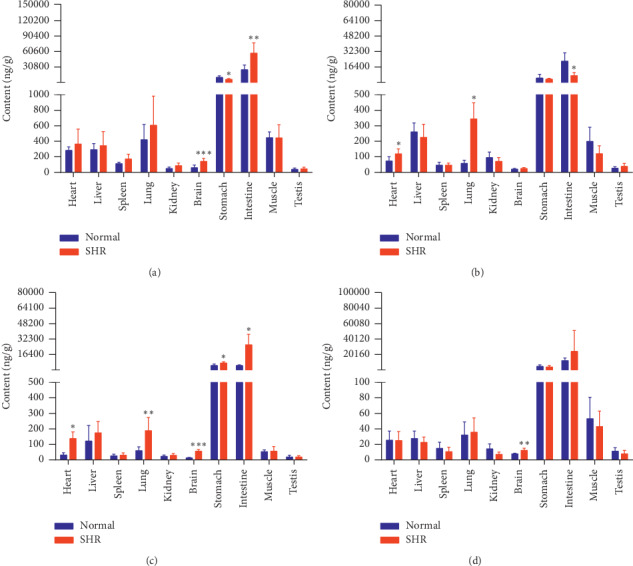
Content of PDG in different rat tissue homogenates of the normal and SHR models at four different points in time after intragastric administration of EC extract (mean ± SD, *n* = 5). (a) 10 min, (b) 30 min, (c) 2 h, and (d) 8 h.

**Figure 14:**
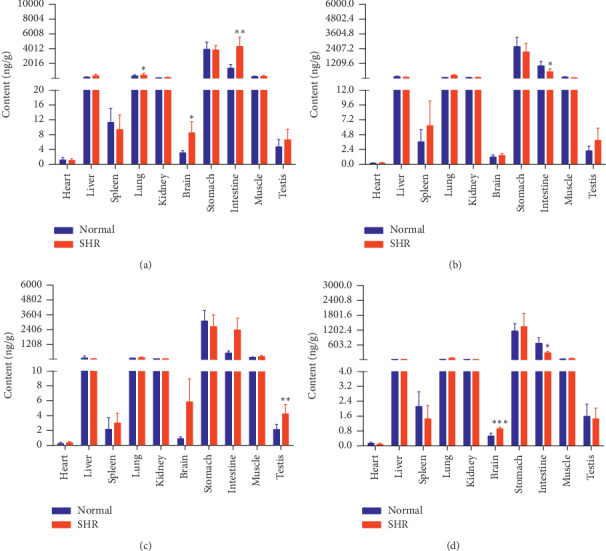
Content of PG in different rat tissue homogenates of the normal and SHR models at four different points in time after intragastric administration of EC extract (mean ± SD, *n* = 5). (a) 10 min, (b) 30 min, (c) 2 h, and (d) 8 h.

The concentrations of the six active components in both groups of rats at four different time points (10 min, 30 min, 2 h, and 8 h) are shown in Figures 9–14.

Compared with the normal group, GA levels in the hearts, spleens, lungs, and brains of SHRs were higher at 10 min. Levels of GA in these rats' livers, kidneys, and intestines were lower at 30 min. GA levels in the livers, kidneys, and spleens of SHRs were lower at 2 h. Levels of GA in the hearts, livers, spleens, lungs, kidneys, brains, muscles, and testes of SHRs were lower at 8 h. As time progressed, the number of tissues that differed in GA distribution between SHRs and normal rats gradually increased, with GA levels lower in the SHRs.

Compared with the normal group, PCA levels in the hearts, lungs, and brains of SHRs were higher at 10 min. Levels of PCA in the testes and stomachs of SHRs were higher at 30 min. PCA levels in the stomachs and intestines of SHRs were higher at 2 h and lower in these rats' intestines and muscles at 8 h.

Compared with the normal group, SHRs had lower levels of NCA in their kidneys and higher levels of NCA in their brains at 10 min. NCA levels in the stomachs of SHRs were higher at 30 min. Levels of NCA in their stomachs were higher and in their muscles lower, at 2 h. NCA levels in the intestines of SHRs were higher at 8 h.

SHRs had lower levels of CA in their kidneys and higher levels in their brains, lungs, and intestines than the normal group at 10 min. Levels of CA in the stomachs of SHRs were higher at 30 min. CA levels were higher in the stomachs and brains of SHRs and lower in their muscles at 2 h. SHRs had higher levels of CA in their brains and hearts at 8 h. In contrast to NCA levels, levels of CA were, to some extent, similar at 10 min, 30 min, and 2 h between both groups of rats.

PDG levels were higher in the brains and intestines of SHRs and lower in their stomachs at 10 min, compared with the normal group. Levels of PDG in the hearts and lungs of SHRs were higher and that in their intestines lower, at 30 min. SHRs demonstrated higher levels of PDG in their hearts, lungs, brains, stomachs, and intestines at 2 h. Levels of PDG in the brains of SHRs rats were higher at 8 h.

Compared with the normal group, levels of PG in the lungs, brains, and intestines of SHRs were higher at 10 min. PG levels in SHRs' intestines were lower at 30 min. Levels of PG in the testes of SHRs were higher at 2 h. At 8 h, SHRs had higher levels of PG in their brains and lower levels thereof in their intestines. In contrast to levels of PDG at the same time point, the SHR and normal groups had, to some extent, similar PDG levels at 10 min and 30 min.

## 5. Discussion

The phenomenon by which a drug is transported between blood and tissues is called distribution [17]. In oral administration, the drug is first absorbed into blood circulation and then distributed to various tissues, body fluids, and cells throughout the body. The distribution process is usually completed very quickly. That is, a reversible balance is achieved. If the main tissue to which the drug is distributed happens to be the drug's site of action (also called its target tissue), there is a close relationship between drug distribution and drug efficacy. Drug distribution to a nonactive site is often closely related to accumulation of the drug in the body and drug toxicity. Therefore, understanding the *in vivo* distribution characteristics of drugs is of great significance for predicting the drugs' pharmacological effects, as well as degree of *in vivo* retention and toxic side effects, and for ensuring the development of safe new medications [18].

Some researchers have studied *in vivo* content analysis methods for EC [19, 20], but a method for simultaneously detecting multiple components in multiple organs has not yet been reported. In this study, we report an analytical method for evaluating levels of six active ingredients (GA, PCA, CA, NCA, PDG, and PG) of EC extract in the tissues (heart, liver, spleen, lungs, kidneys, brain, intestines, stomach, muscles, and testes) of rats. Furthermore, we systematically verified the methodology from the aspects of specificity, linear range, precision and accuracy, extraction recovery, and stability. Our experimental results demonstrated that this method could be used to simultaneously detect levels of the above mentioned six components in different tissues from rats. Moreover, this method was fast, accurate, and sensitive, thus meeting the requirements of biological-sample detection methods.

Drugs usually act on a pathological body, and the body in the pathological state often affects the pharmacokinetics of the drug to a certain extent [21]. Therefore, tissue distribution of the drug more closely approximates clinical-setting conditions in the pathological state than in the physiological state. For this study, we created a SHR model of hypertension to study the tissue distribution characteristics of six active components of EC extract in physiological and pathological bodies. Levels of these components were detected in the tissues of both kinds of experimental animals at four different points in time. We concluded that the six components were distributed in each type of tissue in both groups of rats, and, moreover, distributed to different degrees between normal rats and SHRs.

GA concentration in SHR hearts decreased faster than in those of normal rats. Except in the liver, the concentration of GA in the SHR model at 10 min was higher than in normal rats. In the kidneys of normal rats, the concentration of GA increased with time, and GA levels in their kidneys were higher than in all their other organs at 8 h. However, GA concentration in SHRs peaked at 10 min and then gradually decreased. We speculate that, of the six compounds, GA probably had the highest accumulation of all in the kidneys of normal rats, but not in those of SHRs. PCA in each organ of animals from both groups reached a maximum concentration at 10 min, gradually decreasing from that point onward. PCA concentrations in the livers and kidneys of normal rats were higher than in those of SHRs at 10 min, while in these rats' other organs, PCA levels were lower than or nearly the same as those in SHRs. From 10 min to 30 min, intestinal concentration of PCA in SHRs decreased to a significantly greater degree than in normal rats. The gradient of CA distribution in each organ in both groups was roughly the same, but at various time points, levels in the brains of SHRs were significantly higher than in those of normal rats. PDG levels in the SHR group were higher than those in normal rats, except that the two groups did not significantly differ in PDG level in their testes and muscles at 10 min, and PDG concentrations in the stomachs of normal rats were higher than in those of SHRs. PDG levels in the hearts of SHRs showed a small fluctuation, while those in the hearts of normal rats decreased significantly. From 10 min to 30 min, PG levels in the intestines and stomachs of SHRs decreased faster than in those of normal rats, and the distribution gradients for the remaining organs were almost the same.

In summary, tissue distribution of the six representative components of EC in the SHR model was quite different from that in normal rats, but the reasons for these differences are still unclear. The concentration of six active ingredients in the blood can affect their distribution of six active ingredients in the tissues. Moreover, male-specific pathogen-free Wistar rats and SHRs were from different strains, which may have affected the absorbance of these six active ingredients into the blood. Therefore, the different characteristics of the distribution of six active ingredients may have been caused by the pathological state or the differences in rat strains. However, the underlying mechanism remains to be further studied. Therefore, in subsequent studies, we will further explore the mechanism by which the pathological state of spontaneous hypertension significantly changes the tissue distribution of the main active ingredients found in EC extract.

## 6. Conclusions

The pathological state of spontaneous hypertension could significantly affect the tissue distribution of active antihypertensive ingredients from an EC extract in rats, which could provide a certain reference value for rational use in hypertensive patients requiring long-term use of EC.

## Figures and Tables

**Table 1:** Blood pressure values of normal rats and SHRs before the tissue distribution experiment (mean ± SD, *n* = 24).

Group	Blood pressure values (mmHg)
Normal rats	119 ± 2.6
SHRs	178.7 ± 4.3^∗^

Compared with the normal group: ^*∗*^*P* < 0.05.

**Table 2:** Regression equations of the analytes in rat heart, liver, spleen, lung, and kidney tissue homogenates.

Tissue	Detection component	Linear equation	*R* ^2^	Linear range (ng/g)	LLOQ (ng/g)
Heart	GA	*Y* = 0.1669*X* − 0.2193	0.9991	2.2171∼567.60	2.2171
	PCA	*Y* = 0.0592*X* − 0.0387	0.9993	3.1218∼799.20	3.1218
	NCA	*Y* = 6.699*X* + 19.35	0.9998	1.5312∼98.00	1.5312
	CA	*Y* = 0.017*X* − 0.005	0.9995	1.5031∼96.20	1.5031
	PDG	*Y* = 0.0154*X* − 0.2704	0.9997	3.5507∼909.00	3.5507
	PG	*Y* = 0.0203*X* + 0.0537	0.9994	1.5312∼98.00	1.5312
Liver	GA	*Y* = 0.4247*X* + 1.5948	0.9992	1.8476∼3784.00	1.8476
	PCA	*Y* = 0.1034*X* − 0.3163	0.9998	3.9029∼1998.00	3.9029
	NCA	*Y* = 0.0371*X* − 0.2492	0.9972	1.5312∼98.00	1.5312
	CA	*Y* = 0.0098*X* − 0.0092	0.9996	1.5031∼96.20	1.5031
	PDG	*Y* = 0.097*X* − 1.3094	0.9997	3.5507∼909.00	3.5507
	PG	*Y* = 0.043*X* − 0.2613	0.9996	1.5312∼980.00	1.5312
Spleen	GA	*Y* = 0.1235*X* − 0.1496	0.9993	1.8476∼946.00	1.8476
	PCA	*Y* = 0.3525*X* + 0.0263	0.9992	3.1218∼99.90	3.1218
	NCA	*Y* = 0.4026*X* + 0.719	0.9998	1.5312∼98.00	1.5312
	CA	*Y* = 0.0088*X* − 0.0016	0.9976	1.5031∼96.20	1.5031
	PDG	*Y* = 0.0136*X* − 0.049	0.9992	3.5507∼181.80	3.5507
	PG	*Y* = 0.0292*X* + 0.0096	0.9993	1.5312∼98.00	1.5312
Lung	GA	*Y* = 0.1289*X* − 5.1599	0.9994	1.8476∼7568.00	1.8476
	PCA	*Y* = 0.1378*X* − 0.1911	0.9991	3.1218∼999.00	3.1218
	NCA	*Y* = 0.4829*X* + 0.15	0.9998	1.9140∼490.00	1.914
	CA	*Y* = 0.0038*X* + 0.0156	0.9972	1.8788∼481.00	1.8788
	CPDG	*Y* = 0.0258*X* + 0.3656	0.9982	3.5507∼909.00	3.5507
	PG	*Y* = 0.0114*X* + 0.1654	0.9981	1.5312∼980.00	1.5312
Kidney	GA	*Y* = 0.2401*X* + 2.3586	0.9997	1.8477∼4730	1.8477
	PCA	*Y* = 0.2475*X* − 2.1839	0.9998	3.1218∼999.00	3.1218
	NCA	*Y* = 0.0204*X* + 0.047	0.9988	1.5312∼98.00	1.5312
	CA	*Y* = 0.0124*X* + 0.0344	0.999	1.5031∼96.20	1.5031
	PDG	*Y* = 0.023*X* + 0.0055	0.9993	2.8362∼90.90	2.8362
	PG	*Y* = 0.0333*X* − 0.0443	0.9997	1.7754∼454.5	1.7754

**Table 3:** Regression equations of the analytes in rat brain, small intestine, stomach, muscle, and testis tissue homogenates.

Tissue	Detection component	Linear equation	*R* ^2^	Linear range (ng/g)	LLOQ (ng/g)
Brain	GA	*Y* = 0.1117*X* + 0.0235	0.9996	1.4781∼378.40	1.4781
	PCA	*Y* = 0.1255*X* − 0.0119	0.9996	3.1218∼99.90	3.1218
	NCA	*Y* = 0.5686*X* + 0.2097	0.9996	1.5312∼98.00	1.5312
	CA	*Y* = 0.0257*X* + 0.014	0.9984	1.5031∼96.20	1.5031
	PDG	*Y* = 0.0127*X* − 0.0558	0.9992	3.5507∼181.80	3.5507
	PG	*Y* = 0.0225*X* − 0.0055	0.9995	1.5312∼98.00	1.5312
Intestine	GA	*Y* = 0.234*X* − 6.9843	0.9997	1.7321∼7095.00	1.7321
	PCA	*Y* = 0.2018*X* − 1.6418	0.9995	3.9029∼499.50	3.9029
	NCA	*Y* = 0.0114*X* − 0.0022	0.9996	1.5312∼98.00	1.5312
	CA	*Y* = 0.0052*X* + 0.006	0.9985	1.5031∼96.20	1.5031
	PDG	*Y* = 0.2196*X* + 90.757	0.9996	3.5507∼22725.00	3.5507
	PG	*Y* = 0.0111*X* + 0.0342	0.9992	1.5312∼7840.00	1.5312
Stomach	GA	*Y* = 0.003*X* + 0.0851	0.9992	1.8477∼4730.00	1.8477
	PCA	*Y* = 0.0078*X* − 0.0039	0.9995	1.8477∼7568.00	1.8477
	NCA	*Y* = 0.0156*X* + 0.1213	0.9989	1.5312∼98.00	1.5312
	CA	*Y* = 0.006*X* + 0.0544	0.9985	1.5031∼96.20	1.5031
	PDG	*Y* = 0.0009*X* + 0.0058	0.9998	3.5507∼36360.00	3.5507
	PG	*Y* = 0.0116*X* + 0.147	0.9995	1.5312∼7840.00	1.5312
Muscle	GA	*Y* = 0.0065*X* − 0.0031	0.9997	1.4781∼378.4	1.4781
	PCA	*Y* = 0.0139*X* + 0.086	0.9982	2.2968∼294.00	2.2968
	NCA	*Y* = 0.0028*X* + 0.0035	0.9991	1.9140∼1960.00	1.914
	CA	*Y* = 0.0021*X* + 0.0079	0.9992	1.8788∼1924.00	1.8788
	PDG	*Y* = 0.0146*X* − 0.1305	0.9999	3.5507∼909.00	3.5507
	PG	*Y* = 0.0231*X* − 0.0737	0.9982	1.5312∼392.00	1.5312
Testis	GA	*Y* = 0.0072*X* − 0.0275	0.9993	1.9140∼245.00	1.914
	PCA	*Y* = 0.021*X* − 0.0082	0.9997	3.1219∼99.90	3.1219
	NCA	*Y* = 0.0308*X* − 0.0062	0.9996	1.5312∼98.00	1.5312
	CA	*Y* = 0.0211*X* − 0.0002	0.998	1.5031∼96.20	1.5031
	PDG	*Y* = 0.0135*X* − 0.0142	0.9997	2.8406∼90.90	2.8406
	PG	*Y* = 0.0208*X* − 0.0017	0.9994	1.5312∼98.00	1.5312

## Data Availability

The data used to support the findings of this study are available from the corresponding author upon reasonable request.
